# A randomised open-label pilot trial comparing mycophenolate mofetil with no immunosuppression in limited cutaneous systemic sclerosis (MINIMISE-Pilot)

**DOI:** 10.1093/rheumatology/keag108

**Published:** 2026-02-24

**Authors:** Christopher P Denton, Philip Yee, Medha Kanitkar, Hannah Sims, Charlotte Clarke, Saiam Ahmed, Voon H Ong, Francesco Del Galdo, John D Pauling, Marina E Anderson, Muditha Samaranayaka, Michael Hughes, Smita Bhat, Bridget Griffiths, Maya H Buch, Ariane L Herrick, David D’Cruz, Madelon C Vonk, Nick Freemantle, Hakim-Moulay Dehbi

**Affiliations:** Division of Medicine, University College London, London, UK; Royal Free London NHS Foundation Trust, London, UK; Royal Free London NHS Foundation Trust, London, UK; Royal Free London NHS Foundation Trust, London, UK; UCL Comprehensive Clinical Trials Unit, UCL Institute of Clinical Trials and Methodology, University College London, London, UK; UCL Comprehensive Clinical Trials Unit, UCL Institute of Clinical Trials and Methodology, University College London, London, UK; UCL Comprehensive Clinical Trials Unit, UCL Institute of Clinical Trials and Methodology, University College London, London, UK; MRC Clinical Trials Unit at UCL, UCL Institute of Clinical Trials and Methodology, University College London, London, UK; Division of Medicine, University College London, London, UK; Royal Free London NHS Foundation Trust, London, UK; Leeds Institute of Rheumatic and Musculoskeletal Medicine, University of Leeds, Leeds, UK; North Bristol NHS Trust, Bristol, UK; Lancaster University & NHS University Hospitals of Liverpool Group, Liverpool, UK; Salford Royal Hospital, Northern Care Alliance NHS Foundation Trust, Salford, UK; Salford Royal Hospital, Northern Care Alliance NHS Foundation Trust, Salford, UK; Centre for Musculoskeletal Research, University of Manchester and NIHR Manchester Biomedical Research Centre, Manchester University NHS Foundation Trust, Manchester, UK; Ninewells Hospital and Medical School, Dundee, UK; Freeman Hospital Newcastle, Newcastle, UK; Centre for Musculoskeletal Research, University of Manchester and NIHR Manchester Biomedical Research Centre, Manchester University NHS Foundation Trust, Manchester, UK; Salford Royal Hospital, Northern Care Alliance NHS Foundation Trust, Salford, UK; Centre for Musculoskeletal Research, University of Manchester and NIHR Manchester Biomedical Research Centre, Manchester University NHS Foundation Trust, Manchester, UK; Guy’s and St Thomas’ Hospitals NHS Foundation Trust, London, UK; Department of Rheumatology, Radboud University Medical Center, Nijmegen, The Netherlands; UCL Comprehensive Clinical Trials Unit, UCL Institute of Clinical Trials and Methodology, University College London, London, UK; UCL Comprehensive Clinical Trials Unit, UCL Institute of Clinical Trials and Methodology, University College London, London, UK

**Keywords:** mycophenolate mofetil, immunosuppression, feasibility pilot, limited cutaneous systemic sclerosis, outcome measure, patient reported outcome, autoantibody, centromere

## Abstract

**Objectives:**

Mycophenolate mofetil (MMF) is routinely used in early diffuse cutaneous systemic sclerosis (dcSSc) but not in limited cutaneous (lc)SSc. This may miss an opportunity to slow disease progression. MINIMISE-Pilot tested the feasibility of an open-label event-driven randomised trial of MMF *vs* no immunosuppression in lcSSc.

**Methods:**

We tested the feasibility of a trial evaluating the impact of MMF on a novel event-driven composite endpoint. The MINIMISE endpoint measures time to worsening of lcSSc determined by progressive lung fibrosis, pulmonary hypertension, scleroderma renal crisis, heart failure, severe gut involvement, major digital vascular complications or death. Prespecified ‘Stop–Go’ criteria were agreed. Subjects were stratified by ACA status.

**Results:**

Recruitment was challenging. A total of 53 subjects were screened and 43 were randomised, 21 to the MMF arm. Since recruitment was <60 participants, MINIMISE-Pilot was terminated based upon the prespecified threshold for continuation. During the treatment period there were no clinical worsening endpoints. Adherence to MMF was generally high, with 19 participants (95%) being 100% adherent at week 1, decreasing to 9 participants (64%) at week 24.

**Conclusion:**

MINIMISE-Pilot achieved its goal as a feasibility trial, leading to early termination of the study due to low recruitment. The rationale and concept for this study remain very strong. However, our findings suggest that a randomised prospective trial across 12 sites in the UK with relatively short follow-up duration is not feasible. This will inform the design of future studies testing the benefit of MMF in lcSSc.

**Trial registration:**

Eudract (https://eudract.ema.europa.eu/) 2019-004139-21

Rheumatology Key messages

- We report a feasibility external pilot trial evaluating mycophenolate mofetil in limited cutaneous systemic sclerosis.
- A novel MINIMISE endpoint of ‘time to clinical worsening of systemic sclerosis’ is proposed.
- Recruitment was challenging: our results do not support a definitive full trial without modification.

## Introduction

Systemic sclerosis (SSc) has a high unmet need due to non-lethal burden and severe internal organ disease that can be life threatening [1]. Operationally, SSc is classified according to the extent or severity of skin involvement into two major subsets. Compared with diffuse cutaneous SSc (dcSSc), limited cutaneous SSc (lcSSc) is defined by skin involvement restricted to the distal limbs, face and neck, with no skin thickening proximal to the elbows and knees or extending to the chest or abdomen [2]. It is the most frequent subset, accounting for more than two-thirds of patients in most centres. Any of the major organ–based complications can occur in lcSSc, although generally less frequently except for pulmonary hypertension and gastrointestinal tract disease, which are similar across subsets [3, 4].

SSc has a complex pathogenesis including autoimmunity, vasculopathy and fibrosis. Persistent tissue injury and perturbed repair or regeneration is central to the disease and underlies its pathology and clinical impact. Immunosuppression is routinely started in dcSSc for skin involvement. Although there has never been a prospective randomised controlled trial of mycophenolate mofetil (MMF) in SSc, there is a growing body of evidence supporting its use and efficacy. Indeed, it is a first-line treatment recommended in recent guidelines from the BSR [5] and recommendations from the EULAR [6]. This is based upon observational cohort studies, extrapolation of results from comparative clinical trials in lung fibrosis and most recently from a large analysis of high-quality data from a randomised placebo-controlled clinical trial in dcSSc that permitted background immunosuppression [7]. Subgroup analyses of the large SENSCIS trial (NCT02597933) of SSc-associated interstitial lung disease (SSc-ILD) demonstrated smaller declines in lung function among patients treated with MMF at baseline compared with those who were not, both in the overall study population and in the subset of patients with lcSSc, irrespective of treatment allocation [8, 9].

In this context we sought to explore potential benefit of MMF in lcSSc to determine whether this might prevent development of clinically meaningful complications of the disease. This was predicated on the hypothesis that reported improvements in survival and outcome for dcSSc may have arisen due in part to early use of MMF for skin involvement that may benefit future organ-based complications [10]. Recent studies of SSc have confirmed autoantibody profiles are strongly associated with prognosis and the risk of complications. For example, patients with anti Scl-70 antibodies show the same time and risk of development of interstitial lung fibrosis early in their disease independent of skin subset [4]. Conversely, ACA, which is frequently seen in lcSSc but only occasionally in dcSSc, is associated with a lower risk of interstitial lung fibrosis and generally a better long-term survival across both subsets [11].

The potential for early immunosuppression to modify the disease course of SSc makes an event-driven study attractive. It allows aggregation of clinically meaningful events that occur across subsets. This is analogous to the use of time to clinical worsening in studies of pulmonary arterial hypertension, which rapidly led to convincing clinical trial data for emerging therapies [12, 13]. We therefore have previously proposed a novel composite endpoint of time to clinical worsening for SSc and have applied this to the limited cutaneous subset [14].

The goal of MINIMISE-Pilot was to test the feasibility of a multicentre UK study across key SSc centres using a randomised open-label design. The primary aim was to assess recruitment feasibility, while secondary outcomes included evaluating event rates for the novel time-to-SSc clinical worsening endpoint and examining safety, tolerability and acceptability of MMF among patients with lcSSc.

## Methods

### Study design

MINIMISE-Pilot was a multicentre, phase II, randomised prospective open-label external pilot trial comparing MMF at the standard dose with no immunosuppression in adults with lcSSc. The primary objective was to assess recruitment feasibility and endpoint viability in an event-driven trial requiring relatively infrequent visits that reflect normal follow-up and management of lcSSc. Specific ‘Stop–Go’ criteria to inform a future full MINIMISE trial were defined to assist with interpretation of the study (Supplementary Fig. S1). The target for full recruitment of MINIMISE-Pilot was 120 subjects; <60 subjects would trigger early termination of the study. The trial was conducted in compliance with the approved protocol, the Declaration of Helsinki (2008) and the principles of Good Clinical Practice. All participants provided written informed consent.

Subjects were randomly allocated 1:1 to either an active treatment group receiving MMF or to a control group receiving no active immunosuppression. Randomisation used minimisation with a random element to reduce predictability, balancing for ACA positivity and disease duration.

### Patients

Male and female patients ≥18 years of age with a diagnosis of SSc fulfilling 2013 EULAR and ACR classification criteria [15] and classified to the limited cutaneous subset were eligible [2]. Duration from the first non-Raynaud’s manifestation of SSc could not exceed 7 years. Women of childbearing potential had to have a negative pregnancy test at screening and baseline visits. Key exclusion criteria included having already developed a complication of SSc requiring initiation of MMF or an alternative major immunosuppressive drug such as methotrexate, cyclophosphamide or azathioprine. Additionally, any active viral or bacterial infection was an exclusion criterion. Suitability for enrolment after recovery from infection was based on the investigator’s judgment. Clinically significant abnormalities in haematological or biochemical blood tests, including renal and liver function, were not permitted.

### Treatment

Participants randomised to the active treatment group received a target dose of 2 g of MMF daily for the duration of the study (up to 96 weeks) or until reaching a ‘clinical worsening’ study endpoint. MMF dose was started at 1 g daily and increased over the first 4 weeks post-randomisation to achieve the 2 g target dose, in the absence of side effects. All participants enrolled in the trial received background standard-of-care medical therapy for SSc-related symptoms.

### Outcomes

This study focused on feasibility outcomes that recorded recruitment rate (the proportion of eligible participants enrolled into the trial), adherence to the protocol and the proportion of participants intolerant to MMF who discontinue therapy or reduce their dose. Estimates of the number of centres and length of recruitment period required, identification of barriers to recruitment and assessment of withdrawal rates due to adverse events, as well as loss to follow-up, were provided to inform the design of a future definitive double-blind placebo-controlled trial. The safety and tolerability of MMF in lcSSc was determined by number and type of adverse events, serious adverse events and serious adverse reactions. Clinical outcomes included a novel MINIMISE endpoint of ‘time to clinical worsening of lcSSc’. This was defined as development of new progressive lung fibrosis, pulmonary hypertension, scleroderma renal crisis, heart failure, severe gut involvement causing malnutrition, major vascular complications in the fingers such as gangrene or mortality. Criteria for each component of the composite outcome are defined in Supplementary Data S1.

Secondary clinical endpoints included the change in relevant SSc endpoints of modified Rodnan skin score (mRSS), Patient self-Assessment of Skin Thickness in the Upper Limb (PASTUL) [16], Scleroderma Skin Patient-Reported Outcome (SSPRO) [17], Scleroderma Health Assessment Questionnaire (SHAQ) and health-related quality of life by EuroQol 5-dimension 5-level (EQ-5D-5L) questionnaire, including pain and disability. Separate patient and physician’s global assessment scales were also collected [18, 19].

### Statistical analysis

As this was an external pilot trial designed to assess feasibility, no formal inferential statistical analyses were performed. Descriptive statistics were used to summarise cohort characteristics and to inform sample size estimation and power calculations for a future full trial. Prespecified ‘go’ and ‘no go’ criteria were defined regarding recruitment and event rates. These helped with interpretation of the data from MINIMISE-Pilot.

## Results

### Patient recruitment

**Figure 1:**
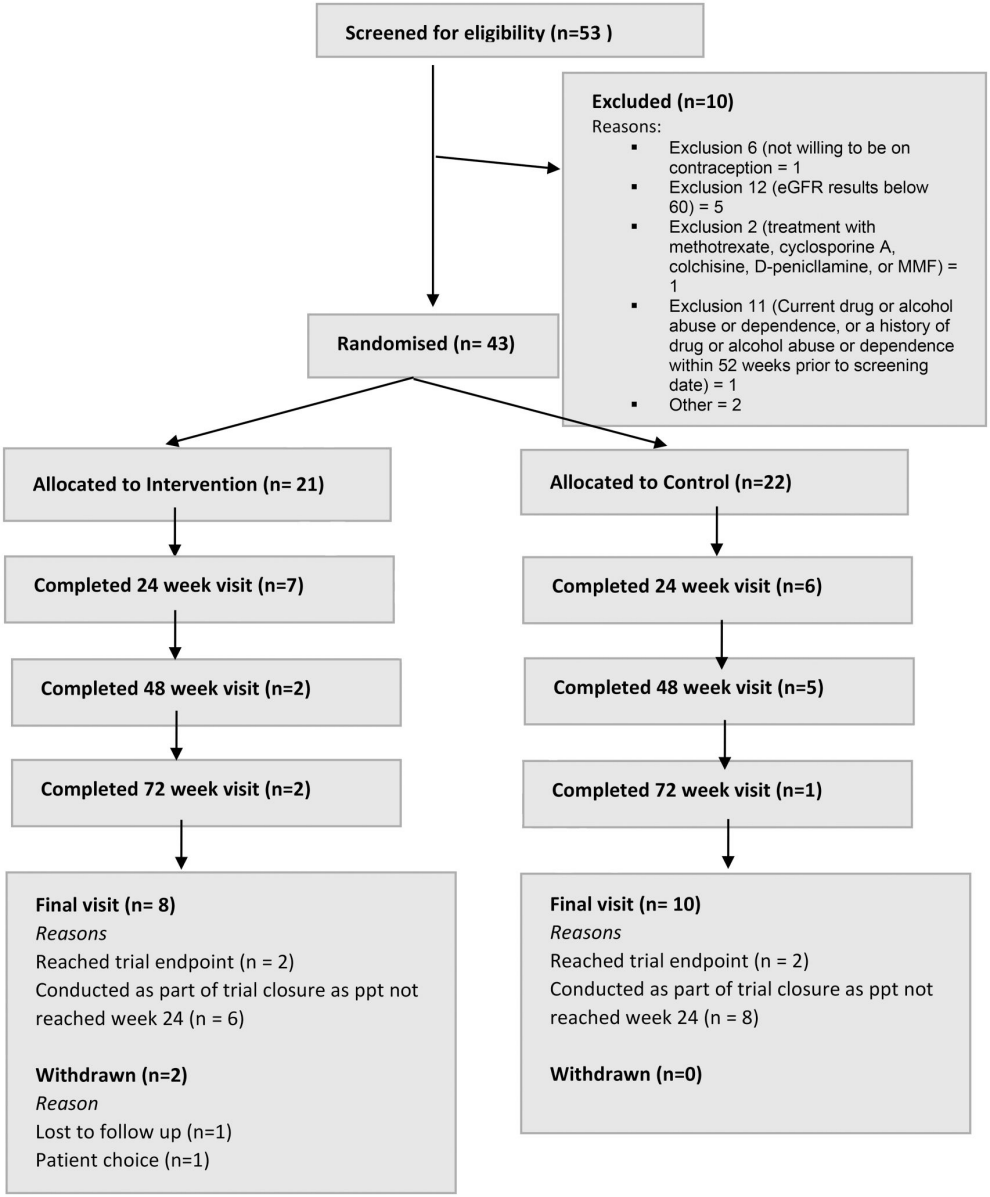
CONSORT diagram showing patient disposition for MINIMISE-Pilot. Graphical summary of the actual recruitment figures for MINIMISE-Pilot showing subjects assessed for eligibility, excluded, randomised, allocated and followed up

Recruitment and patient disposition are summarised in a CONSORT diagram (Fig. 1). A total of 53 patients were screened for eligibility and 10 patients were excluded: 1 of childbearing potential was unwilling to use contraception, 5 had an estimated glomerular filtration rate (eGFR) <60 ml/min/1.73 m^2^, 1 was receiving other immunosuppressive treatment, 1 had alcohol dependence and 2 were excluded for other reasons. A total of 43 subjects were randomised: 21 to receive immunosuppression with MMF and 22 to receive placebo, each in addition to standard-of-care treatment.

As this was a feasibility pilot study, the rate of recruitment and site activation were carefully compared with projected rates based upon estimates made prior to commencement of the trial. The actual and projected recruitment rates are shown graphically in Supplementary Fig. S2. Thus, although the target number of patients were screened and enrolled at the lead site, recruitment was substantially below the target across other participating sites. MINIMISE-Pilot was terminated based upon the prespecified threshold for continuation in the ‘Stop–Go’ criteria since recruitment was <60 participants (Supplementary Fig. S1). It is noteworthy that although up to 13 recruiting sites had been anticipated, not all were activated due to local delays and logistical issues (Supplementary Fig. S2).

### Characteristics of study participants

Clinical and demographic features of the study cohort are summarised in Table 1 for the overall cohort and by treatment allocation. Most patients were female (84%) and White British (81%), mean age was 56.56 years (s.d. 9.20) and average eGFR was 76.43 ml/min/1.73 m^2^ (s.d. 11.87). Thirty-one patients were ACA positive (14 MMF, 17 control). The median upper limb mRSS was 2.0 [interquartile range (IQR) 4.0], median PASTUL score was 4.0 (IQR 5.0) and median HAQ disability index (HAQ-DI) was 0.69 (IQR 0.94).

**Table 1:** Baseline characteristics: demographics and clinical.

Baseline characteristics	Control (*n* = 22)	MMF (*n* = 21)	Total (*N* = 43)
Age (years), mean (s.d.)	57.14 (10.71)	55.95 (7.51)	56.56 (9.20)
Sex, *n* (%)			
Female	19 (86.36)	17 (80.95)	36 (83.72)
Male	3 (13.64)	4 (19.05)	7 (16.28)
Ethnicity, *n* (%)			
White: White British	19 (86.36)	16 (76.19)	35 (81.40)
White: White (other background)	0 (0)	3 (14.29)	3 (6.98)
White: White Irish	2 (9.09)	0 (0)	2 (4.65)
Asian or Asian British: Indian	1 (4.55)	0 (0)	1 (2.33)
Black or Black British: African	0 (0)	1 (4.76)	1 (2.33)
Black or Black British: Black (other background)	0 (0)	1 (4.76)	1 (2.33)
ACA status, *n* (%)			
Positive	17 (77.27)	14 (66.67)	31 (72.09)
Negative	5 (22.73)	7 (33.33)	12 (27.91)
Disease duration (years), *n* (%)			
<4	16 (72.73)	14 (66.67)	30 (69.77)
≥4	6 (27.27)	7 (33.33)	13 (30.23)
Echocardiogram result, *n* (%)			
Abnormal: not clinically significant	1 (4.55)	2 (9.52)	3 (6.98)
Within normal limits	14 (63.64)	12 (57.14)	26 (60.47)
Missing	7 (31.82)	7 (33.33)	14 (32.56)
Pulmonary function test result, *n* (%)			
Abnormal: clinically significant	1 (4.55)	2 (9.52)	3 (6.98)
Abnormal: not clinically significant	3 (13.64)	4 (19.05)	7 (16.28)
Within normal limits	15 (68.18)	10 (47.62)	25 (58.14)
Missing	3 (13.64)	5 (23.81)	8 (18.60)
ECG result, *n* (%)			
Abnormal: not clinically significant	2 (9.09)	3 (14.29)	5 (11.63)
Within normal limits	15 (68.18)	17 (80.95)	32 (74.42)
Missing	5 (22.73)	1 (4.76)	6 (13.95)

### Safety

There were no major safety concerns. A total of 104 adverse events occurred during the study: 26 (25%) in the control arm and 78 (75%) in the MMF arm. Classification by system organ class is provided in Table 2. Serious adverse events (SAEs) occurred in three patients (7%). These included two (9%) in the control group and one (5%) in the MMF group. SAEs reported included gastrointestinal tract disorders (one MMF) requiring hospital admission, one (control) injury and procedural complication and one (control) worsening of connective tissue disease. Adherence to MMF was generally high, with 19 participants (95%) being 100% adherent at week1, decreasing to 9 participants (64%) at week 24. During the treatment period no participants experienced meaningful worsening of clinical endpoints. No adverse events linked to the COVID-19 pandemic were observed [20].

**Table 2:** Safety assessment: AEs and SAEs by system organ class.

AEs	Control (*n* = 26)	MMF (*n* = 78)	Total (*N* = 104)
MedDRA system organ class, *n* (%)			
Cardiac disorders	0 (0)	1 (1.28)	1 (0.96)
Eye disorders	0 (0)	3 (3.85)	3 (2.88)
Gastrointestinal disorders	5 (19.23)	20 (25.64)	25 (24.04)
General disorders and administration site conditions	2 (7.69)	12 (15.38)	14 (13.46)
Hepatobiliary disorders	0 (0)	1 (1.28)	1 (0.96)
Immune system disorders	0 (0)	1 (1.28)	1 (0.96)
Infections and infestations	7 (26.92)	16 (20.51)	23 (22.12)
Injury, poisoning and procedural complications	3 (11.54)	1 (1.28)	4 (3.85)
Investigations	0 (0)	2 (2.56)	2 (1.92)
Metabolism and nutrition disorders	0 (0)	1 (1.28)	1 (0.96)
Musculoskeletal and connective tissue disorders	0 (0)	3 (3.85)	3 (2.88)
Nervous system disorders	3 (11.54)	8 (10.26)	11 (10.58)
Psychiatric disorders	0 (0)	2 (2.56)	2 (1.92)
Reproductive system and breast disorders	0 (0)	2 (2.56)	2 (1.92)
Respiratory, thoracic and mediastinal disorders	3 (11.54)	2 (2.56)	5 (4.81)
Skin and subcutaneous tissue disorders	3 (11.54)	1 (1.28)	4 (3.85)
Social circumstances	0 (0)	1 (1.28)	1 (0.96)
Vascular disorders	0 (0)	1 (1.28)	1 (0.96)

SAEs, *n* (%)	Control (*n* = 2)	MMF (*n* = 1)	Total (*N* = 3)
MedDRA system organ class			
Gastrointestinal disorders	0 (0)	1(100.00)	1 (33.33)
Injury, poisoning and procedural complications	1 (50.00)	0 (0)	1 (33.33)
Musculoskeletal and connective tissue disorders	1 (50.00)	0 (0)	1 (33.33)

### Clinical outcomes at baseline and follow-up

Clinical outcomes are tabulated showing baseline values for the whole cohort as well as each study arm. Baseline measurements show that the two study arms were generally well matched. Follow-up values are shown for all time points of the study with the number of subjects indicated per study arm and time point. Since the study was terminated prematurely, few data were available for later time points.

Adherence to MMF was generally high, with 19 participants (95%) being 100% adherent at week 1, decreasing to 9 participants (64%) at week 24. The decrease in adherence reflected logistical reasons due to early termination of the study.

Data for skin assessment tools at baseline and follow-up are shown in Table 3. The mean baseline mRSS was higher in the MMF group, and consistent with the lcSSc subset, the average skin score was <5 units. There was no significant change over time in mRSS, allowing for the small number of patients assessed at the final visit, noting that the number assessed who were on MMF was substantially higher. PASTUL was shown to be feasible as an assessment tool for skin involvement in lcSSc. In line with previous observational data, numerically higher mean mRSS in the MMF arm is reflected by PASTUL results. The SSPRO data indicate that the numerically greater average skin involvement in the MMF arm at baseline is reflected in a higher SSPRO score. There is no apparent change in the average SSPRO score over the first two trial visits.

**Table 3:** Skin assessment tools outcome data at baseline and follow-up.

Score	Control, *n*	MMF, *n*	Control, mean (s.d.)	MMF, mean (s.d.)	Estimate (95% CI) of mean difference (MMF *vs* placebo)	*P*-value
MRSS						
Baseline	17	18	2.47 (2.18)	4.11 (3.46)	–	–
Visit 2	7	8	2.29 (1.89)	4.25 (2.71)	0.51 (−1.92, 2.93)	0.691
Visit 3^a^	2	3	4.50 (3.54)	3.33 (2.52)	−1.92 (−6.61, 2.77)	0.506
Visit 4^b^	–	1	–	2.00 (NA)	–	–
Visit 5^b^	–	–	–	–	–	–
Final visit	4	11	2.25 (1.26)	4.09 (3.86)	2.33 (−3.51, 8.17)	0.464
PASTUL						
Baseline	22	21	4.36 (3.03)	5.81 (5.77)	–	–
Visit 2	14	12	4.07 (2.50)	5.75 (5.08)	1.38 (−1.18, 3.94)	0.303
Visit 3	5	4	3.40 (2.97)	6.50 (3.42)	2.56 (−3.36, 8.48)	0.445
Visit 4^a^	2	2	2.50 (0.71)	5.50 (4.95)	13.50 (10.25, 16.75)	0.078
Visit 5^b^	–	1	–	8.00 (NA)	–	–
Final visit	7	10	5.00 (5.69)	5.00 (4.76)	1.08 (−3.49, 5.65)	0.651
SSPRO						
Baseline	22	21	26.23 (24.31)	30.86 (26.59)	–	–
Visit 2	14	12	20.21 (15.54)	32.08 (29.28)	5.05 (−9.06, 19.16)	0.491
Visit 3	5	4	13.60 (15.87)	44.75 (40.58)	14.78 (−44.17, 73.73)	0.649
Visit 4^a^	2	2	36.00 (7.07)	21.00 (7.07)	−5.40 (−23.77, 12.97)	0.667
Visit 5^b^	–	1	–	50.00 (NA)	–	–
Final visit	7	10	40.43 (29.86)	33.20 (32.96)	−5.99 (−26.30, 14.33)	0.574

NA: not applicable.

^a^Estimated mean difference in score between treatment and placebo adjusted only for baseline score.^b^No estimate due to one or no observations at time point. ^c^Unadjusted estimated mean difference in score between treatment and placebo.

The results for the SHAQ self-assessment tool are shown in Table 4. HAQ-DI scores were comparable between the MMF and control arms at baseline. Scores increased slightly from baseline to visit 2 in both arms, but there was no statistically significant difference between treatment arms at any time point. The SHAQ includes five SSc-specific and one global impact visual analogue scale (VAS). Overall, these scores were generally comparable between the two arms. However, lung scores were consistently higher and worsened over follow-up among those allocated to MMF. Pain and gastrointestinal scores also worsened considerably at week 3 among those in the MMF arm. Baseline Raynaud’s symptoms were substantially worse in patients allocated to MMF, which was reflected in higher ulcer VAS scores. Overall disease assessment was very similar at baseline for both study groups.

**Table 4:** SHAQ data.

Score	Control, *n*	MMF, *n*	Control, mean (s.d.)	MMF, mean (s.d.)	Estimate (95% CI) of mean difference (MMF *vs* placebo)	*P*-value
HAQ-DI						
Baseline	21	21	0.70 (0.63)	0.85 (0.68)	–	
Visit 2	12	12	0.86 (0.56)	0.89 (0.71)	−0.10 (−0.38, 0.16)	0.457
Visit 3	6	4	0.46 (0.49)	0.81 (0.88)	0.15 (−0.39, 0.69)	0.608
Visit 4^a^	2	2	0.69 (0.27)	0.88 (0.18)	0.19 (−0.31, 0.68)	0.592
Visit 5^b^	–	–	–	–	–	–
Final visit	7	11	0.95 (0.67)	0.93 (0.79)	−0.21 (−0.37, −0.05)	0.027
SHAQ pain VAS						
Baseline	19	19	31.21 (28.61)	30.53 (28.72)	–	–
Visit 2	12	12	28.17 (32.79)	28.08 (23.55)	−2.29 (−27.39, 22.81)	0.860
Visit 3^a^	6	3	39.33 (27.52)	50.33 (10.02)	9.90 (−69.23, 89.02)	0.829
Visit 4^c^	2	2	20.00 (19.80)	55.50 (4.95)	35.50 (7.21, 63.78)	0.133
Visit 5^b^	–	–	–	–	–	–
Final visit	7	11	56.86 (32.96)	37.00 (28.46)	−27.64 (−69.04, 13.77)	0.220
SHAQ GI VAS						
Baseline	20	19	26.25 (31.93)	29.05 (36.64)	–	–
Visit 2	12	12	24.75 (28.27)	48.17 (41.04)	18.99 (−10.15, 48.14)	0.220
Visit 3	6	3	20.50 (27.60)	60.67 (44.74)	67.53 (6.25, 128.80)	0.276
Visit 4^c^	2	2	31.50 (28.99)	50.50 (57.28)	19.00 (−69.97, 107.97)	0.716
Visit 5^b^	–	–	–	–	–	–
Final visit	7	11	73.86 (34.93)	25.27 (36.68)	−57.47 (−98.93, −16.01)	0.022
SHAQ lung VAS						
Baseline	20	19	17.35 (22.82)	23.47 (31.23)	–	–
Visit 2	12	12	19.75 (32.03)	28.00 (31.04)	1.99 (−22.00, 25.97)	0.873
Visit 3	6	3	21.67 (25.96)	40.33 (35.50)	37.50 (8.64, 66.36)	0.238
Visit 4^c^	2	2	4.50 (3.54)	50.00 (59.40)	45.50 (−36.97, 127.97)	0.393
Visit 5^b^	–	–	–	–	–	–
Final visit	7	11	33.00 (42.47)	29.82 (34.40)	−17.34 (−53.87, 19.18)	0.374
SHAQ Raynaud’s VAS						
Baseline	18	19	22.11 (22.82)	44.58 (36.49)	–	–
Visit 2	12	12	32.08 (29.39)	35.33 (32.52)	0.20 (−32.99, 33.40)	0.991
Visit 3^a^	6	3	37.83 (35.56)	57.00 (50.76)	55.50 (39.25, 71.75)	0.094
Visit 4^c^	2	2	47.00 (25.46)	51.50 (68.59)	4.50 (−96.90, 105.90)	0.939
Visit 5^b^	–	–	–	–	–	–
Final visit	7	11	43.00 (35.11)	29.18 (35.06)	−21.07 (−43.45, 1.31)	0.095
SHAQ ulcer VAS						
Baseline	20	19	8.30 (20.31)	14.53 (30.75)	–	–
Visit 2	12	12	1.50 (3.18)	21.17 (28.93)	12.07 (−3.41, 27.55)	0.146
Visit 3	6	3	20.33 (31.53)	22.00 (32.97)	−32.52 (−207.75, 142.71)	0.778
Visit 4^c^	2	2	0.00 (0.00)	3.00 (1.41)	3.00 (1.04, 4.96)	0.095
Visit 5^b^	–	–	–	–	–	–
Final visit	7	11	7.43 (17.92)	17.09 (28.49)	−0.75 (−27.76, 26.25)	0.958
SHAQ disease VAS						
Baseline	19	19	28.58 (25.76)	27.89 (21.90)	–	–
Visit 2	12	12	29.92 (27.41)	33.42 (23.11)	−1.62 (−23.51, 20.27)	0.887
Visit 3	6	3	27.00 (21.67)	52.67 (34.44)	64.92 (40.39, 89.45)	0.121
Visit 4^c^	2	2	61.00 (29.70)	58.50 (30.41)	−2.50 (−61.41, 56.41)	0.941
Visit 5^b^	–	–	–	–	–	–
Final visit	7	11	54.71 (33.09)	32.00 (32.60)	−52.59 (−92.02, −13.16)	0.026

^a^Estimated mean difference in score between treatment and placebo adjusted only for baseline score. ^b^No estimate due to one or no observations at time point. ^c^Unadjusted estimated mean difference in score between treatment and placebo.

Global and quality-of-life assessments are shown in Table 5 and provide additional insights into the impact of lcSSc. Baseline EQ-5D-5L scores were very similar between the two arms. Patient global and physician global assessments were very similar between treatment arms at baseline and showed consistency at visit 2. Beyond this time point, the number of data points available was too small to interpret. In line with the statistical analysis plan, no formal interpretation can be drawn from these differences; however, they provide useful information about baseline characteristics and potential for change over time in future studies of lcSSc.

**Table 5:** Quality of life and global assessment data at baseline and follow-up.

Score	Control, *n*	MMF, *n*	Control, mean (s.d.)	MMF, mean (s.d.)	Estimate (95% CI) of mean difference (MMF vs placebo)	*P*-value
EQ-5D-5L						
Baseline	21	21	0.78 (0.13)	0.77 (0.20)	–	–
Visit 2	14	12	0.74 (0.11)	0.73 (0.19)	0.01 (−0.08, 0.10)	0.824
Visit 3	5	4	0.83 (0.12)	0.64 (0.19)	−0.13 (−0.31, 0.05)	0.226
Visit 4^a^	2	2	0.75 (0.05)	0.72 (0.02)	−0.14 (−0.40, 0.13)	0.497
Visit 5^b^	–	–	–	–	–	–
Final visit	7	11	0.68 (0.13)	0.78 (0.19)	0.14 (0.02, 0.25)	0.039
EQ-5D-5L VAS						
Baseline	21	21	64.38 (20.13)	70.48 (22.63)	–	–
Visit 2	14	12	64.07 (17.20)	60.17 (22.65)	−2.97 (−17.15, 11.22)	0.686
Visit 3	5	4	62.00 (9.08)	62.50 (15.55)	1.29 (−10.01, 12.59)	0.834
Visit 4^a^	2	2	50.00 (14.14)	52.50 (10.61)	1.60 (−47.24, 50.43)	0.959
Visit 5^b^	–	–	–	–	–	–
Final visit	7	11	48.43 (22.45)	61.18 (31.79)	11.91 (−18.27, 42.10)	0.454
Patient Global Assessment						
Baseline	21	21	44.57 (23.38)	41.43 (24.76)	–	–
Visit 2	14	11	38.86 (20.00)	41.27 (23.85)	−6.03 (−20.68, 8.61)	**0.429**
Visit 3	5	3	29.80 (24.77)	55.00 (25.98)	−16.42 (−73.83, 40.99)	**0.632**
Visit 4^a^	2	2	42.50 (3.54)	60.00 (28.28)	−5.30 (−6.55, −4.04)	**0.077**
Visit 5^b^	–	–	–	–	–	–
Final visit	7	11	50.00 (25.17)	33.00 (31.67)	−25.37 (−45.12, −5.62)	**0.026**
Physician’s Global Assessment						
Baseline	22	21	27.91 (19.10)	25.86 (19.22)	–	–
Visit 2	12	11	27.33 (19.37)	28.18 (20.03)	−1.44 (−16.22, 13.34)	0.851
Visit 3	4	3	32.50 (22.17)	38.33 (22.55)	6.72 (−25.77, 39.21)	0.724
Visit 4^a^	2	3	15.00 (7.07)	28.33 (2.89)	14.15 (6.40, 21.91)	0.070
Visit 5^b^	–	–	–	–	–	–
Final visit	8	12	28.75 (27.48)	14.50 (11.96)	−16.91 (−31.21, −2.60)	0.035

^a^Estimated mean difference in score between treatment and placebo adjusted only for baseline score. ^b^No estimate due to one or no observations at time point difference in score between treatment and placebo.

It is notable that there were no MINIMISE events observed, most likely due to the smaller number of subjects and shorter follow-up than expected.

## Discussion

Here we report the results of a pilot study to evaluate the feasibility of a multicentre trial across UK scleroderma centres randomising patients with lcSSc to receive MMF at a standard dose or no immunosuppression. The main goals of the study were to assess the ease of recruitment and the viability of a novel event-driven clinical endpoint for lcSSc. Prespecified criteria for a lack of feasibility based on recruitment were met and the study was terminated early. No MINIMISE composite endpoint events occurred, probably due to the small number of patients recruited and short duration of follow-up, so the viability of this endpoint cannot be concluded. It is notable that the endpoint has performed well in several recent cohort studies and can differentiate subgroups of lcSSc, as detailed below.

There were no major safety concerns and MMF was generally well tolerated. The adverse event profile is in line with experience in current clinical practice and other recent clinical trials. During the study, MMF emerged as a recommended first-line treatment for skin in dcSSc and as a cornerstone for managing SSc-ILD in both disease subsets. MMF use for dcSSc is supported by a growing evidence base [21], among which is a recent post hoc analysis of the RESOLVE-1 clinical trial (NCT03398837) [22]. Although no randomised placebo-controlled trial of MMF has been conducted in SSc, findings from observational analyses and extrapolation of results from other studies, including a large multicentre clinical trial, consistently support the efficacy of MMF as a treatment for both skin and lung disease [5, 6, 21, 22].

Unfortunately, our findings do not support extrapolation of the current study design from this external pilot to a full clinical trial, largely due to much lower rates of recruitment than anticipated across most study sites. It is notable that the lead centre successfully met its recruitment target. This suggests operational aspects of the study and eligibility criteria were feasible despite substantial problems at other sites. Thus future effort should consider how other sites can be supported to optimise and maximise recruitment and overcome local hurdles to activation and recruitment. One particular challenge was related to funding ‘excess treatment costs’ within the current National Health Service (NHS) and National Institute for Health Research (NIHR) landscape. Although modest within the context of the overall study budget and resources, the treatment costs were not included as research costs and so had to be supported locally at the sites. Since there was no simple mechanism for central funding of the excess treatment costs, this caused major delays in finalising the study agreement at some sites. This is something that should be avoided in the future if studies are to recruit in an efficient and timely manner.

The COVID-19 pandemic had a substantial operational impact on MINIMISE-Pilot. The final protocol and regulatory approvals were all in place and ready to start the study early in 2020. This coincided with the first wave of the COVID-19 pandemic in the UK, leading to a national lockdown and redeployment of clinical staff away from research and into clinical care to deal with the pandemic. Continuing with the trial at the start of the pandemic was impossible, so it was paused and later recommenced once patient visits were possible to the study sites. It is highly likely that some of the challenges in trial set-up, recruitment and management were a result of the COVID-19 pandemic [20, 23]. Thus it is possible that recruitment targets would have been reached across multiple trial sites without COVID-19. It is possible that there were concerns by potential subjects or treating physicians about risks of immunosuppression from MMF, especially while the pandemic was ongoing. This may explain why some prescreened patients declined participation in the trial.

Clinical trial endpoints and outcome measures are challenging in SSc. Many recent trials have focused on patients with diffuse skin involvement or specific complications such as ILD. Potential outcomes that have been informative in other studies include mRSS, forced vital capacity and composite outcome measures such as the ACR Composite Response Index in Systemic Sclerosis [24, 25]. In lcSSc, an event-driven composite outcome is attractive because it captures clinically meaningful disease progression and aggregates events that are individually infrequent in a cohort. By analogy with complications such as pulmonary arterial hypertension, ‘time to clinical worsening’ is an attractive approach for exploring long-term benefit of treatment. Our strategy and proposed endpoint are supported by recent observational studies that have confirmed the viability of the MINIMISE time-to-event endpoint in observational cohorts, including a large cohort of lcSSc subjects with long-term follow-up at our centre [14, 26]. Stratification using a serum chemokine interferon signature predicts significantly different endpoint rates. Similar data are available related to autoantibody profile within lcSSc and confirming the higher rate of events in ACA-negative lcSSc [4]. Other future stratification might consider vascular treatments of complications of lcSSc. To reduce bias, the preferred study design for a definitive trial would include blinding to treatment allocation.

Although skin involvement is less extensive in lcSSc than in dcSSc, it is still meaningful. Severe skin thickening, even when confined to the distal limbs, face and neck, can be very troublesome. Assessment of skin severity has traditionally used the mRSS or patient-reported symptom questionnaires such as SSPRO [17]. To complement these approaches, we developed PASTUL as a remote assessment tool with patient-reported assessment of skin severity of the upper limb [16]. Recent multicentre validation of PASTUL in an observational study confirms strong correlation with mRSS and other quality-of-life measures and excellent test–retest reliability [27]. By focusing on upper limb involvement, this novel endpoint is particularly suited to patients with lcSSc and PASTUL was included as a novel outcome in the current trial. In MINIMISE-Pilot, PASTUL was shown to be a feasible endpoint and was stable between visits 1 (baseline) and 2, where there were ≥12 subjects assessed in each study arm. Further evaluation is required before PASTUL can be recommended as a primary endpoint in clinical trials, but it is a promising secondary or exploratory endpoint. In combination with the SSPRO, this self-reported measure of skin thickness may provide a comprehensive patient-reported assessment of skin severity for decentralised clinical trials in SSc. Other initiatives to develop composite measures relevant to lcSSc are under way [28, 29].

In conclusion, this was the first prospective clinical trial exclusively recruiting patients with lcSSc and using a novel event-driven endpoint of ‘time to clinical worsening of SSc’. It establishes the rationale for testing MMF in lcSSc using an event-driven design. Recruitment at the lead site was to target but, unfortunately, logistic delays and slower recruitment in other sites do not support this study design for a future definitive full trial. Nevertheless, we consider that collective evidence supports future research to establish whether benefit of routine use of MMF in lcSSc outweighs risk.

## Supplementary Material

keag108_Supplementary_Data

## Data Availability

Appropriate data from this study can be made available for academic research upon reasonable request to the corresponding author.
