# Supplementary material for: A randomised open-label pilot trial comparing mycophenolate mofetil with no immunosuppression in limited cutaneous systemic sclerosis (MINIMISE-Pilot)
Source: Rheumatology (Oxford). 2026 Feb 24;65(3):keag108. doi: 10.1093/rheumatology/keag108 (PMC13032817; doi:10.1093/rheumatology/keag108)
Supplement: keag108_Supplementary_Data [file keag108_supplementary_data.zip › rhe-25-2617-File003.docx]

**Supplementary Figures**

**Supplementary Figure S1 Pre-specified “Stop” and “Go” criteria for MINIMISE-Pilot**

Prior to funding approval there was agreement amongst sponsor, funder and lead applicant about thresholds for “Stop” and “Go” considering progression from this external feasibility pilot towards a full clinical trial using a similar design template. The criteria reflected the small size of this study and that it could not provide any definitive comparative data concerning efficacy. This minimum thresholds for recruitment, event number and numerical values regarding any safety events were calculated. If the results were in the red zone, then this suggested that a similarly designed full trial was not feasible. It in the green zone then a full study could proceed unaltered. The amber zone would prompt revision and adjustment to design for a future full study.

**
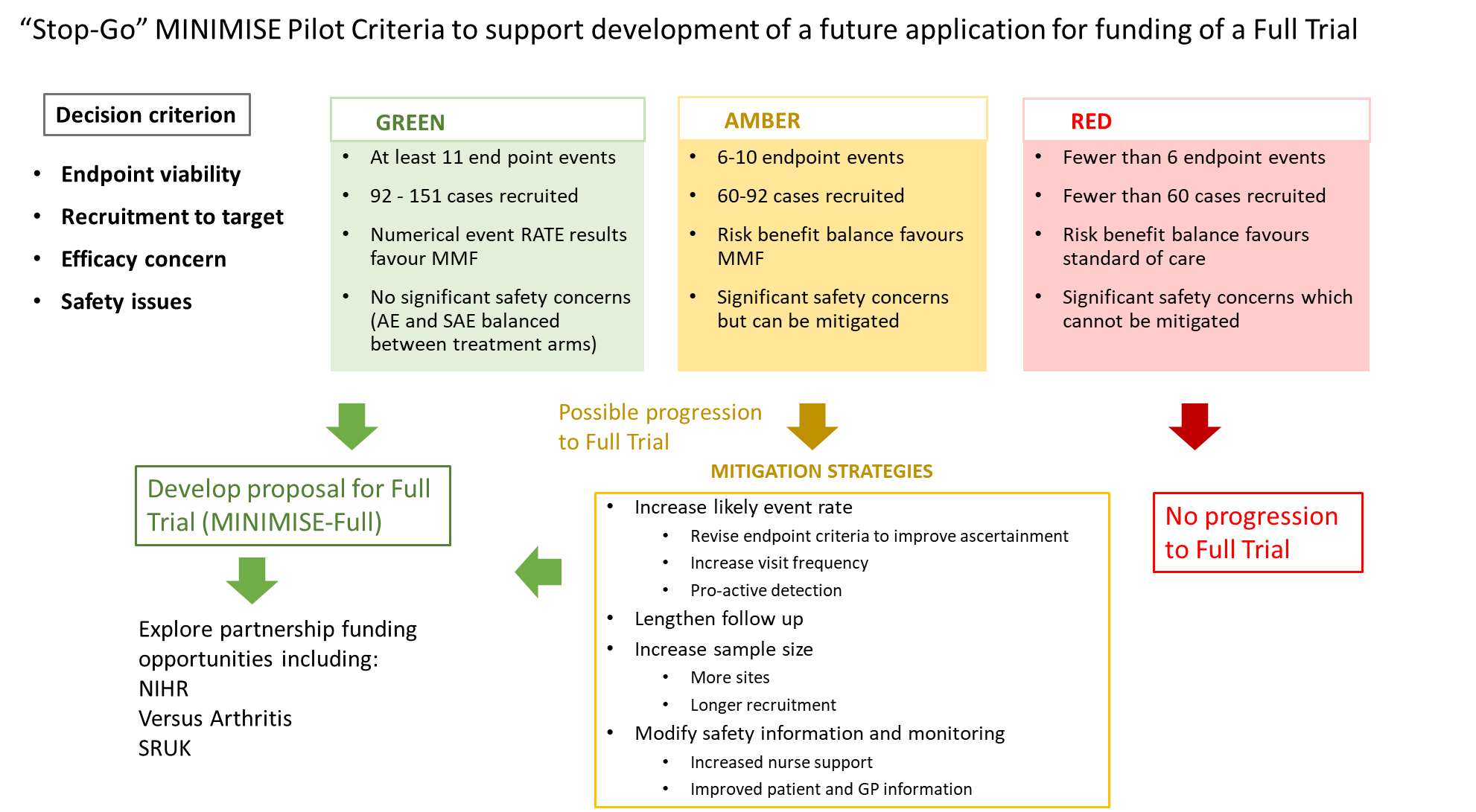
**

**
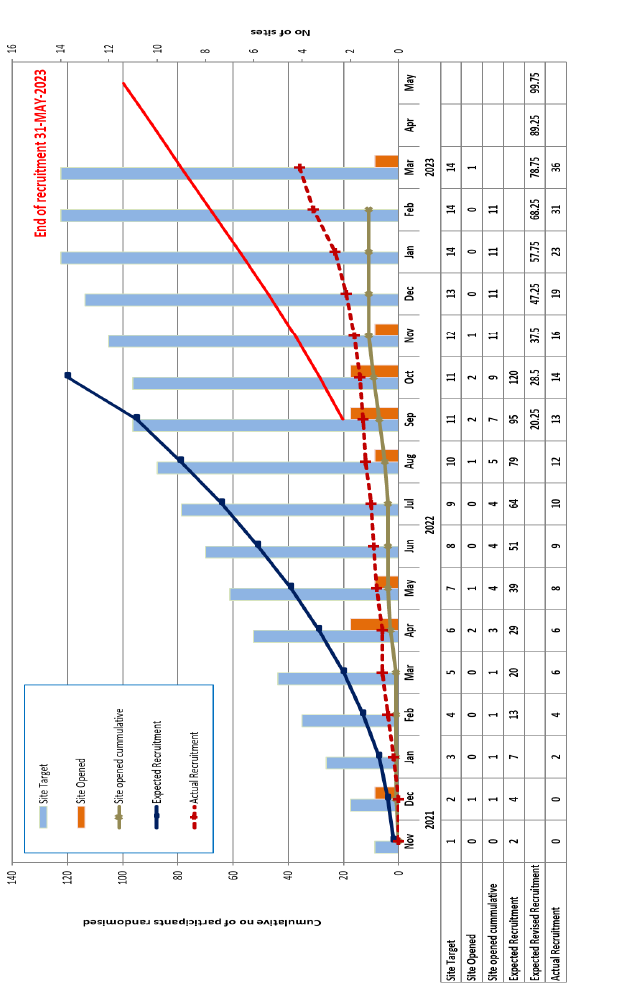
Supplementary Figure S2 Site activation, actual and target recruitment for MINIMISE-Pilot**

Graphical summary of the timeline for site activation and the predicted and actual recruitment of subjects. Although the lead site had excellent recruitment, activation of other sites was generally slow and recruitment much below target leading to early termination of this study once its goal as a feasibility pilot had been achieved by recruitment and event rate being within the “red” zone for prespecified thresholds (Figure S1).
