# Supplementary material for: A randomised open-label pilot trial comparing mycophenolate mofetil with no immunosuppression in limited cutaneous systemic sclerosis (MINIMISE-Pilot)
Source: Rheumatology (Oxford). 2026 Feb 24;65(3):keag108. doi: 10.1093/rheumatology/keag108 (PMC13032817; doi:10.1093/rheumatology/keag108)
Supplement: keag108_Supplementary_Data [file keag108_supplementary_data.zip › rhe-25-2617-File002.docx]

**Supplementary Data S1 – detailed description of the MINIMISE composite endpoint**

**Time to clinical worsening of limited cutaneous systemic sclerosis (lcSSc) defined as development of new clinically significant endpoint:**

The MINIMISE clinical worsening composite endpoint consists of the following:

- New lung fibrosis on HRCT with FVC< 70%. This will require definite CT appearance of lung fibrosis with at least 5% of lung involved and FVC less than 70% predicted with technically acceptable spirometry.
- Deterioration of established lung fibrosis (composite categorical decline (CCD) defined by reduction in FVC by at least 10% or FVC 5-9% with DLco 15% fall [8]). Cases will all have definite lung fibrosis affecting at least 5% of lung fields and changes in lung function will be interpreted only with technically satisfactory measurement. Changes will be absolute % predicted.
- Significant progression of modified Rodnan skin score (MRSS increase of at least 5 units and 25% increase from baseline). Assessment by an assessor proficient in MRSS.
- Haemodynamically significant cardiac complication related to SSc defined by systolic ejection fraction less than 45% or large pericardial effusion impairing cardiac function or arrhythmia requiring anti- arrhythmic therapy (cardioversion, medical or device) or need for a cardiac pacemaker.
- Scleroderma renal crisis as defined by the core items of new proposed classification criteria [see Butler EA, Baron M, Fogo AB, et al. Generation of a Core Set of Items to Develop Classification Criteria for Scleroderma Renal Crisis Using Consensus Methodology. Arthritis Rheumatol. 2019 Jun;71(6):964-971. doi: 10.1002/art.40809.
- Confirmed new diagnosis of pulmonary hypertension by RHC according to the 2019 expert definition (mPAP above 20 mm Hg, PVR above 3 Wood Units).
- SSc related GI disease requiring enteral nutritional supplementation for more than 3 weeks or any parenteral feeding or admission over 72 hours for intestinal obstruction or pseudo-obstruction.
- Severe digital vasculopathy (defined as gangrene, amputation, osteomyelitis or septic arthritis as assessed by the investigator and confirmed by imaging or culture) [see Nihtyanova SI, Brough GM, Black CM, Denton CP. Clinical burden of digital vasculopathy in limited and diffuse cutaneous systemic sclerosis. Ann Rheum Dis. 2008 Jan;67(1):120-3]
- Mortality – death from any cause
